# Cost-effectiveness Analysis of Genetic Testing and Tailored First-Line Therapy for Patients With Metastatic Gastrointestinal Stromal Tumors

**DOI:** 10.1001/jamanetworkopen.2020.13565

**Published:** 2020-09-28

**Authors:** Sudeep Banerjee, Abhishek Kumar, Nicole Lopez, Beiqun Zhao, Chih-Min Tang, Mayra Yebra, Hyunho Yoon, James D. Murphy, Jason K. Sicklick

**Affiliations:** 1 Department of Surgery, Division of Surgical Oncology, University of California, San Diego; 2 Department of Surgery, University of California, Los Angeles; 3 Department of Radiation Medicine and Applied Sciences, University of California, San Diego; 4 Department of Surgery, Division of Colorectal Surgery, University of California, San Diego

## Abstract

**Importance:**

Gastrointestinal stromal tumor (GIST) is frequently driven by oncogenic *KIT* variations. Imatinib targeting of *KIT* marked a new era in GIST treatment and ushered in precision oncological treatment for all solid malignant neoplasms. However, studies on the molecular biological traits of GIST have found that tumors respond differentially to imatinib dosage based on the *KIT* exon with variation. Despite this knowledge, few patients undergo genetic testing at diagnosis, and empirical imatinib therapy remains routine. Barriers to genetic profiling include concerns about the cost and utility of testing.

**Objective:**

To determine whether targeted gene testing (TGT) is a cost-effective diagnostic for patients with metastatic GIST from the US payer perspective.

**Design, Setting, and Participants:**

This economic evaluation developed a Markov model to compare the cost-effectiveness of TGT and tailored first-line therapy compared with empirical imatinib therapy among patients with a new diagnosis of metastatic GIST. The main health outcome, quality-adjusted life years (QALYs), and costs were obtained from the literature, and transitional probabilities were modeled from disease progression and survival estimates from randomized clinical trials of patients with metastatic GIST. Data analyses were conducted October 2019 to January 2020.

**Exposure:**

TGT and tailored first-line therapy.

**Main Outcomes and Measures:**

The primary outcome was QALYs and cost. Cost-effectiveness was defined using an incremental cost-effectiveness ratio, with an incremental cost-effectiveness ratio less than $100 000/QALY considered cost-effective. One-way and probabilistic sensitivity analyses were conducted to assess model stability.

**Results:**

Therapy directed by TGT was associated with an increase of 0.10 QALYs at a cost of $9513 compared with the empirical imatinib approach, leading to an incremental cost-effectiveness ratio of $92 100. These findings were sensitive to the costs of TGT, drugs, and health utility model inputs. Therapy directed by TGT remained cost-effective for genetic testing costs up to $3730. Probabilistic sensitivity analysis found that TGT-directed therapy was considered cost-effective 70% of the time.

**Conclusions and Relevance:**

These findings suggest that using genetic testing to match treatment of *KIT* variations to imatinib dosing is a cost-effective approach compared with empirical imatinib.

## Key Points

**Question:**

Is targeted gene testing cost-effective in patients with metastatic gastrointestinal stromal tumor?

**Findings:**

This economic evaluation developed a Markov model to examine the cost-effectiveness of targeted gene testing with tailored imatinib dosing for patients with gastrointestinal stromal tumor based on *KIT* exon variation status. The model found that targeted gene testing was cost-effective, with an incremental cost-effectiveness ratio of $92 100, compared with empirical treatment with imatinib.

**Meaning:**

These findings support the cost-effectiveness of widespread adoption of genetic testing for patients newly diagnosed with metastatic gastrointestinal stromal tumor.

## Teaser

This economic evaluation examines the cost-effectiveness of targeted gene testing for tailored first-line treatment among patients with gastrointestinal stromal tumors (GIST).

## Introduction

Gastrointestinal stromal tumor (GIST) is the most common sarcoma and is frequently driven by oncogenic *KIT* (OMIM 164920) variations. In the late 1990s, the use of targeted therapy against *KIT* variations with imatinib marked a new era in GIST treatment and ushered in precision oncological treatment for all solid malignant tumors.^1^ Although treatment of advanced GIST with imatinib achieved sustained objective responses, primary and acquired secondary resistance to imatinib remains a clinical challenge.^2^ In the setting of imatinib failure, newer generations of tyrosine kinase inhibitors have shown efficacy as second-line (ie, sunitinib) and third-line (ie, regorafenib) agents.^3,4^

Studies on the molecular characteristics of GIST have shown that the disease is genetically diverse. Of approximately 70% of patients with *KIT* variations, 67% have variations implicating exon 11, 10% to 15% have alterations in exon 9, while exons 17, 18, and 13 account for 1% of patients each.^2^ Additionally, other genetic drivers, including *PDGFRA* (OMIM 173490), RAS pathway activation (eg, *KRAS* [OMIM 190070], *HRAS* [OMIM 190020], *NRAS* [OMIM 164790], *BRAF* [OMIM 164757], and *NF1* [OMIM 613113]), succinate dehydrogenase complex deficiency, *ETV6/NTRK3* (OMIM 191316) fusions, and *FGFR1* (OMIM 136350) fusions have also been implicated.^2,5,6,7,8,9,10^ These subgroups differ not only in terms of genetics but also in terms of clinical features, route of metastasis, patient outcomes, and imatinib responsiveness.

With metastatic GIST, the underlying genetic driver influences the probability of treatment success. The MetaGIST trial^11^ was a pooled meta-analysis of 2 large, randomized clinical trials comparing the effectiveness of low-dose (400 mg) vs high-dose (800 mg) imatinib. The MetaGIST trial^11^ used variation analysis performed in both trials and identified *KIT* exon 9 variation as a factor associated with clinical response to high-dose imatinib and with superior recurrence free survival and equivalent overall survival (OS). The National Comprehensive Cancer Network guidelines subsequently recommended testing to identify patients with this variation to ensure initiation of the appropriate starting dose.^12^

Despite this recommendation, only 15% to 33% of patients undergo genetic testing at diagnosis, and 400 mg imatinib is a mainstay of first-line therapy for all GIST.^13,14^ Barriers to genetic profiling may include concerns about the cost and utility of testing. To investigate this, we evaluated the cost-effectiveness of variation profiling for patients with metastatic GIST.

## Methods

The design and reporting of this cost-effectiveness analysis follow standard guidelines published elsewhere.^15^ Analysis of deidentified published data was granted institutional review board and informed consent exemption by the institutional review board of the University of California, San Diego. We adhered to the Consolidated Health Economic Evaluation Reporting Standards (CHEERS) reporting guideline for reporting economic evaluations. Data analyses were conducted October 2019 to January 2020.

### Model Parameters

**Figure 1.**
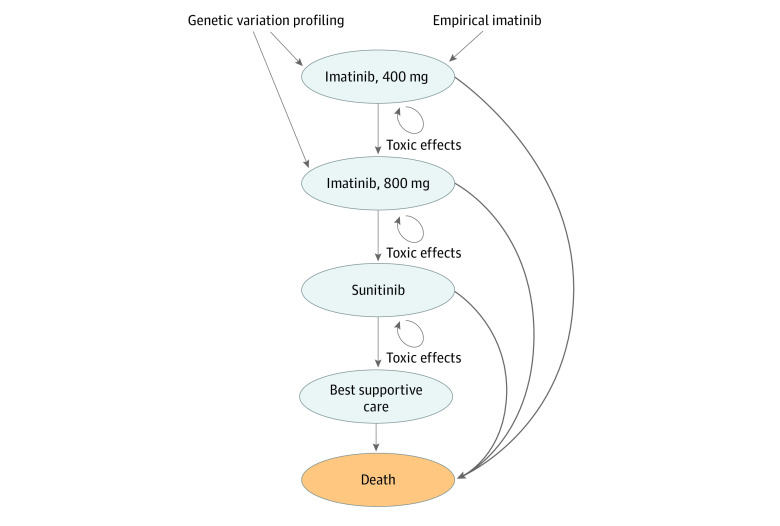
State Transition Diagram All patients were started in imatinib 400 mg as first-line therapy except patients with *KIT* exon 9 variation in the TGT-direct therapy group who were advanced to imatinib 800 mg as first-line therapy. Patients had a risk of death within each health state. Patients had a chance of drug toxic effects within each health state.

We developed a Markov model to compare the cost-effectiveness of targeted gene testing (TGT) and variation-directed first-line therapy compared with empirical imatinib therapy for patients with metastatic GIST from the US payer perspective.^16^ We modeled outcomes for 3 genomic subpopulations: *KIT* exon 11, *KIT* exon 9, and all other variations, based on 2 previous randomized clinical trials.^17,18^ The model simulated treatment outcomes, including progression and death, associated with treatment using first-, second-, and third-line therapy for each genomic subpopulation, following the treatment algorithm defined by the National Comprehensive Cancer Network guideline recommended therapy for metastatic GIST.^12^ Patients within the empirical imatinib group started with low-dose imatinib (ie, 400 mg) as first-line therapy. Disease progression prompted dose escalation to high-dose imatinib (ie, 800 mg). The second occurrence of disease progression was treated with sunitinib. The third occurrence of disease progression was considered treatment failure and patients received best supportive care. In the TGT-directed approach, patients with *KIT* exon 9 variations started on high-dose imatinib as first-line therapy followed by sunitinib and best supportive care in the setting of disease progressions. Patients with *KIT* exon 11 and other variations followed the same treatment algorithm as those in the empirical imatinib group (Figure 1). Treatment with regorafenib was not included owing to the unavailability of clinical trial data for the 3 genomic subpopulations studied in the model. Cycle length was defined as 2 months, based on the National Comprehensive Cancer Network recommendation for assessment of disease progression, and the model time horizon was 10 years. Markov models were constructed and analyzed using TreeAge Pro 2019 (TreeAge Software).

### State Transition Probabilities

Disease progression and OS were modeled based on prospective, randomized clinical trial data for treatment with imatinib or sunitinib in patients with metastatic disease.^11,17,18^ Disease progression and OS were extracted from Kaplan-Meier curves from each trial using WebPlotDigitizer data extraction tool version 4.2,^19^ and transition probabilities for progression and risk of death were modeled for each genomic subpopulation (ie, *KIT* exon 11, *KIT* exon 9, and other variations) and drug treatment combination (ie, imatinib 400 mg, imatinib 800 mg, and sunitinib). Data were extracted from 2 studies: imatinib data was obtained from the secondary analysis of a 2006 phase III randomized clinical trial by Debiec-Rychter et al^17^ while sunitinib data were obtained from a 2008 phase I/II randomized clinical trial by Heinrich et al.^18^ Model transition probabilities were validated by comparison with source clinical trial data for progression free survival (eFigure 1 in the Supplement). Transition probabilities for OS were based on the first-line data for a given Markov node because of model simulation (eFigure 2 in the Supplement). Estimates for OS were based on clinical trial data for the first 3 years, and long-term survival (ie, 3 to 10 years) was estimated from the conditional monthly probability of death of patients with metastatic GIST within the Surveillance, Epidemiology, and End Results database.^20^ The probability of adverse events to imatinib or sunitinib therapy were obtained from reported rates in clinical trials.^21,22^

### Utility Estimates

Effectiveness was measured in quality-adjusted life years (QALYs), which estimates a patient’s health utility over time. Health utility measures quality of life on a scale that ranges from 0 (death) to 1 (perfect health). Health utilities for this study were defined previously using prospective EQ-5D surveys^23,24,25^ (Table). The health utility for a patient with metastatic GIST was defined as 0.935 while the health utility of a patient with metastatic GIST on best supportive care was defined as 0.577.^23,25^ Three opportunities for disease progression (first-line, second-line, and third-line treatments) were included in the model and were associated with healthy utility decrease of 0.12 for each disease progression.^23,25^ There was no QALY deduction associated with toxic effects from imatinib or sunitinib therapy based on clinical trial data that indicated equivalent quality of life before and after treatment with either medication.^3,30^

**Table.** Model Parameters

Parameter	Value (95% CI)	Distribution	Source
**Costs per y, $**^a^			
Targeted gene testing	2919 (1903 to 4150)^b^	γ	Medicare^c^
Drug			
Imatinib, mg			
400	57 690 (38 064 to 85 446)	γ	NADAC, Jabbour et al, 2019^26^
800	115 380 (76 128 to 170 892)	γ	NADAC, Jabbour et al, 2019^26^
Sunitinib	86 726 (51 516 to 114 756)	γ	NADAC
Best supportive care	9403 (5856 to 13 194)	γ	Chabot et al, 2008^23^
Other medical costs			
Imatinib	2315 (1500 to 3252)	γ	Wilson et al, 2005^24^ and Hislop et al, 2011^25^
Sunitinib	3344 (2178 to 4704)	γ	Wilson et al, 2005^24^ and Hislop et al, 2011^25^
Adverse events, No,			
Imatinib	296 (1164 to 2526)^b^	γ	Wu et al, 2017^27^
Sunitinib	160 (612 to 1392)^b^	γ	Remák et al, 2008^28^
Disease progression	46 548 (29 754 to 66 636)^d^	γ	Guerin et al, 2014^29^
**Probabilities**			
Adverse events, per mo^e^			
Imatinib, mg			
400	0.033 (0.021 to 0.047)	β	Verweij et al, 2004^21^
800	0.065 (0.042 to 0.094)	β	Verweij et al, 2004^21^
Sunitinib	0.031 (0.020 to 0.043)	β	Reichardt et al, 2015^22^
Health utilities			
Metastatic GIST	0.935 (0.82 to 1)	β	Wilson et al, 2005^24^ and Hislop et al, 2011^25^
Disease progression	−0.12 (−0.15 to −0.08)	β	Chabot et al, 2008^23^ and Hislop et al, 2011^25^

Abbreviations: GIST, gastrointestinal stromal tumor; NADAC, National Average Drug Acquisition Cost.

^a^ All costs were inflation adjusted to 2019 using the Consumer Price Index Inflation Calculator.

^b^ One-time cost.

^c^ Medicare reimbursement for Targeted Genomic Sequencing Analysis (*Current Procedural Terminology* code 81455).

^d^ Cost applied during first 3 years of model.

^e^ Monthly rate derived from overall clinical trial rate of adverse events.

### Cost Estimates

Costs were estimated using a US payer perspective (Table). The cost of TGT was obtained from Medicare claims data for multigene next-generation sequencing diagnostic tests. Annual costs of imatinib and sunitinib therapy were estimated from the National Average Drug Acquisition Cost (NADAC) database^31^ with a standard 7% reduction. The base case cost of imatinib was a weighted mean of the cost of brand-name and generic imatinib based on the current estimated market share of 50% for generic and 50% for brand-name.^26^ The cost of best supportive care was estimated for patients with treatment refractory metastatic GIST.^23^ The cost of disease progression was based on estimated health care utilization cost of patients with recurrent GIST.^29^ The cost of disease progression was applied in the first 3 years of the model. Costs of adverse events were obtained from clinical trials.^27,28^ All costs were adjusted for inflation to 2019 US dollars using the consumer price index.

### Statistical Analysis

An annual discounting rate of 3% was applied for cost and effectiveness. Cost-effectiveness was determined based on the incremental cost-effectiveness ratio (ICER), which is the ratio of the difference in costs between the 2 treatments divided by the difference in QALYs. For this study, we used a willingness-to-pay threshold of $100 000 per QALY, with treatments less than $100 000 per QALY considered cost-effective. We used 1-way sensitivity analyses to identify individual variables that influenced the cost-effectiveness results. A probabilistic sensitivity analysis was also conducted to explore model parameter uncertainty in all transition probabilities, costs, and health utilities using a Monte Carlo simulation with 100 000 iterations. Cost estimates were modeled with γ distributions and transition probabilities, and health utilities were modeled with β distributions. We obtained SDs for the probability of toxic effects, progression, and death from the literature. The SDs of cost and health utilities were assumed to be 20% of the mean,^32^ and varying this number from 10% to 40% in a sensitivity analysis did not impact results of the probabilistic sensitivity analysis (eTable 1 in the Supplement).

## Results

### Base Case Results

In the base case analysis, TGT-directed therapy was associated with an increase in cost of $9513, from $469 106 with the empirical imatinib approach to $478 619 with TGT-directed therapy. The total accrued QALYs increase by 0.10 with TGT-directed therapy, from 4.88 with empirical imatinib to 4.98 with TGT-directed therapy, and TGT-directed therapy was associated with an ICER of $92 100 per QALY compared with the empirical imatinib approach, which was considered cost-effective at a $100 000 per QALY willingness-to-pay threshold.

### One-Way Sensitivity Analysis

**Figure 2.**
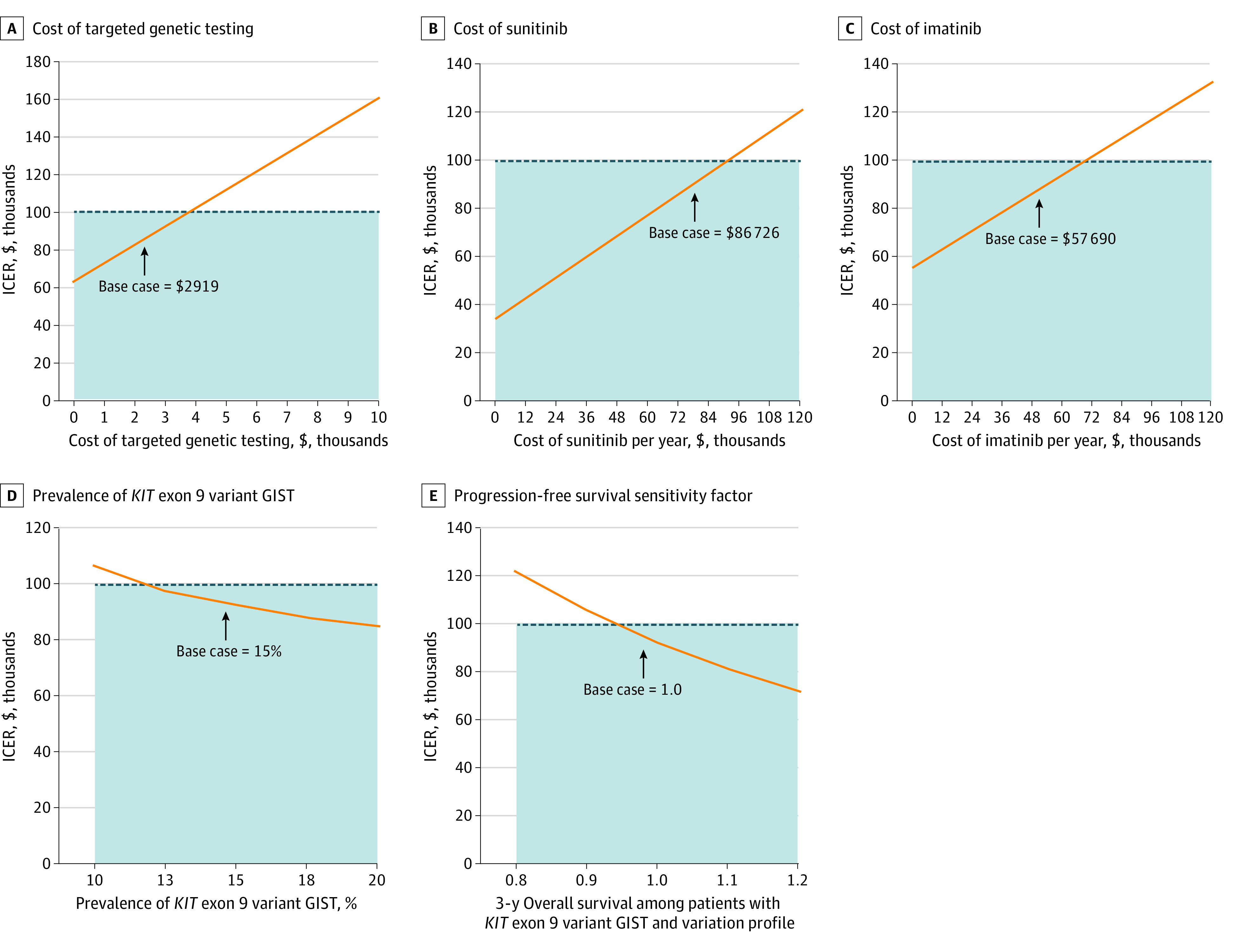
One-Way Sensitivity Analysis Dotted line indicates willingness-to-pay threshold $100 000; orange line, incremental cost-effectiveness ratio (ICER); and shaded region, range of costs within willingness-to-pay threshold.

One-way sensitivity analyses were performed for all variables within the model (eTable 2 in the Supplement). We found that TGT-directed therapy remained cost-effective at a threshold of $100 000 per QALY for TGT costs up to $3730, 28% greater than the cost estimate within the base case (Figure 2A). Furthermore, TGT-directed therapy remained cost-effective at sunitinib costs less than $91 620, a 6% increase from the estimated base case value (Figure 2B). The cost of imatinib per year would have to be $69 924 for TGT-directed therapy to no longer be cost-effective, reflecting an increase by 21% from the base case value (Figure 2C). Sensitivity to the cost of imatinib was further assessed with the assumption that either generic or brand-name drug comprised 100% of market share. In the case of all–brand-name imatinib, the ICER increased to $126 300 per QALY, which was not cost-effective. Alternatively, in the case of all generic imatinib, the ICER decreased to $58 000 per QALY, which was highly cost-effective. We next tested model sensitivity to the prevalence of *KIT* exon 9 variations and found that TGT-directed therapy remained cost-effective if the prevalence was greater than 11.7%, which is 3.3% less than the base case estimate (Figure 2D). In addition, the model was sensitive to the modeled transition probabilities for survival. Overall survival sensitivity analysis revealed that TGT-directed therapy remained cost-effective with 3-year OS estimates less than 51.6% for patients with the *KIT* exon 9 genotype (Figure 2E). Lastly, we tested model sensitivity to the time horizon of the model. At 3 years, the ICER was $151 900, 5 years was $118 800, 10 years was $92 100 (base case), and 15 years was $81 200.

### Probabilistic Sensitivity Analysis

**Figure 3.**
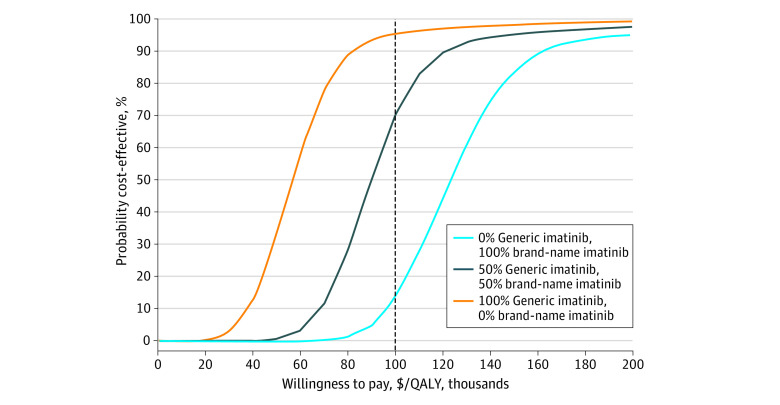
Probabilistic Sensitivity Analysis Dotted line indicates willingness-to-pay threshold.

Probabilistic sensitivity analysis demonstrated the relative stability of our cost-effectiveness analysis results. In the base scenario in which the market share of generic imatinib was 50%, we found that TGT would be cost-effective 69.9% of the time at a willingness-to-pay threshold of $100 000 per QALY (Figure 3). If the market share of generic imatinib was 100%, we found that TGT would be cost-effective 95.1% of the time. Conversely, if the market share of brand name imatinib was 100%, we found that TGT would be cost-effective only 13.5% of the time.

## Discussion

Gastrointestinal stromal tumor (GIST) is often driven by oncogenic variations in *KIT*. While some patients respond to the usual starting dose of imatinib, approximately 10% to 15% of patients harbor a *KIT* exon 9 variation and require high dose imatinib for clinical benefit.^11^ However, only 15% of patients with GIST undergo variation profiling before treatment initiation.^13^ This economic evaluation is the first, to our knowledge, to report a Markov model assessing the cost-effectiveness of using TGT to tailor imatinib therapy in patients with metastatic GIST. Our findings suggest that TGT profiling and personalized imatinib dosing is a cost-effective approach for tailoring the first-line treatment of patients with metastatic GIST. Sensitivity analysis revealed that lower costs of TGT or drugs increased the cost-effectiveness of TGT-directed therapy. Probabilistic sensitivity analysis indicated that TGT-directed therapy would be cost-effective 70% of the time. These findings support a role for TGT before treatment initiation in patients with metastatic GIST.

There are few studies on the cost-effectiveness of gene testing and tailored therapy. One study by Li et al^33^ compared the use of a single *BRAF* V600 variation test to a panel of 34 genes in the diagnostic workup for metastatic melanoma and found that the panel was less expensive and yielded more QALYs than the single-gene test. In a 2017 study on lung cancer,^34^ multiplex targeted sequencing for fourth-line agent selection was cost-ineffective, with an ICER of $485 199 per QALY. Similarly, in a 2016 study on platinum-resistant ovarian cancer,^35^ compared with chemotherapy, TGT and targeted therapy was not cost-effective, with an ICER of $479 303 per QALY. In the latter 2 studies, high ICERs were driven by the significant costs of targeted therapy while both models were relatively insensitive to the cost of TGT. This is also true in our study, in which the cost of TGT represents a small fraction of the overall cost of treatment. However, the clinical scenario tested in this model differs from the these studies because it did not entail a recommended treatment that is an expensive novel agent. Additionally, it is interesting to note that clinical trials and meta-analysis, which were used in this model, show a difference in progression-free survival but not OS for patients with *KIT* exon 9 variations treated with high-dose imatinib compared with those treated with low-dose imatinib. This indicates the QALY gain associated with the TGT-directed therapy approach is entirely derived from reduced incidence of tumor progression. The importance of progression-free survival has been shown across a variety of cancer types and is associated with a clinically relevant deterioration in quality of life.^36^ For these reasons, the cost-effectiveness of withholding futile therapy for patients with *KIT* exon 9 variation is not surprising.

We found the cost of the TGT-directed approach to be $9513 higher than the empirical imatinib approach. This increased cost reflects a subset of patients with *KIT* exon 9 alterations who were treated with more expensive therapy (high-dose imatinib and sunitinib) compared with the least expensive option (ie, low-dose imatinib). This is also the primary reason why improved survival in sensitivity analyses actually rendered the TGT-directed approach less cost-effective. The cost of imatinib is peculiar—costs of the brand-name drug consistently rose in the years leading up to and after the release of generic imatinib.^37^ The reasons for this paradoxical trend are not clear but may in part be owing to delays in the availability of a low cost generic imatinib.^37^ However, recently the brand-name drug has consistently lost market share to the generic option.^38^ Our results demonstrate that TGT-directed therapy could become progressively more cost-effective as generic imatinib continues to comprise a larger market share.

Clinically available TGT diagnostic panels vary by read breadth and depth. The least costly of these usually involve focused gene panels, while whole-exome diagnostics are generally more expensive. Retail cost for these diagnostic tests range from several hundred dollars to more than $10 000. This analysis found that a TGT cost of $3730 or less would be considered cost-effective, although many of these more expensive tests would not be cost-effective when considering their list price. However, the Centers for Medicare & Medicaid Services provides reimbursement for TGT panels that profile approximately 300 or more cancer-related genes. Several commonly used clinical panels fall within this coverage and would be cost-effective in our model.

Several studies have examined the cost-effectiveness of whole-exome and whole-genome assays on diagnostic yield for neurological or neurodevelopmental disorders.^39,40^ These studies emphasize that early implementation of TGT is critical to achieving the cost-effectiveness benefit. This finding complements the clinical situation of treating GIST, since patients who initially do not respond to empirical imatinib may undergo TGT before initiating second-line treatment. Our model would suggest that the addition of TGT after the failure of first-line therapy is dramatically less cost-effective than prior to first-line therapy.

In a 2016 study on variation analysis in GIST, Schöffski et al^41^ estimated cost savings associated with withholding empirical imatinib from patients with a *PDGFRA* D842V variation, which is known to have primary imatinib resistance. Schöffski et al^41^ found that variation profiling and selectively withholding therapy in this genomic subpopulation was cost-saving. In our study, we focused on the value of TGT results to identify patients who harbor a *KIT* exon 9 variation, which can inform dose selection for starting imatinib therapy. However, TGT diagnostics yield a variety of actionable information. Well-implemented TGT data for patients with GIST can prevent trials of empirical imatinib therapy in patients who have genetic alterations known to be intrinsically resistant to imatinib. For example, patients with *PDGFRA* D842V do not respond to imatinib, but preclinical and early clinical trial data have shown excellent results with new targeted therapies.^42^ Patients with *KIT* exon 18 variations or succinate dehydrogenase complex deficiency are also known not to respond to imatinib and can be allocated to alternative TKI therapies, repeated surgical resection or clinical trials.^17^ In this study, we chose to focus on the only 3 genomic subpopulations with robust clinical trial data (ie, *KIT* exon 11, *KIT* exon 9, and all other variations) to optimize model simulation. As a consequence, management differences among the all other variations group were not addressed. The value of TGT clearly extends beyond identification of *KIT* exon variations; however, lack of availability of large clinical trials precludes cost-effectiveness modeling of these genomic subpopulations.

### Limitations

There are several limitations to this study. First, TGT-guided therapy provides a wide array of actionable information that can lead to patients receiving first-line agents other than low-dose imatinib, which could influence cost-effectiveness of TGT. However, the focus of this study was narrowed to imatinib dose selection for patients with *KIT* exon 9 variations because of the availability of robust clinical trial data that could be used to extrapolate valid transition probabilities for our model. Ideally, additional actionable variations and cognate targeted therapies could be included. However, this is contingent on conducting the appropriate clinical trials to gather the necessary data. Second, many patients who receive imatinib undergo dose reduction after experiencing grade II to III adverse effects. Although dose reduction was not explicitly included in the model, the transition probabilities for progression and OS are based on a patient cohort that underwent dose reductions when necessary. Additionally, it is likely that our model overestimates the cost of imatinib because dose reductions would correspond to lower drug costs. Third, we found that our model was sensitive to the transition probabilities that were modeled from clinical trial data. However, it is important to note that clinical trials of low- vs high-dose imatinib permitted crossover for patients in the low-dose imatinib group. This suggests that the estimated rate of survival may be higher than the actual rate, particularly in the patients with *KIT* exon 9 variations. Therefore, we anticipate that our transition probabilities are conservative estimates of the differences between treatments. Fourth, cost of imatinib was estimated based on reports of market share data in the literature.^26^ Although we do not have actual data on the proportion of generic imatinib used, we anticipate that this proportion will increase over time, which would support the conclusions of our model. Fifth, patients in our model were not given a chance to discontinue treatment. This decision was made to develop a theoretical data set to compare TGT-directed therapy vs empirical imatinib. Additionally, we anticipate that rates of treatment discontinuation would be similar in each arm of our model and is unlikely to have a significant impact on the ICER. Sixth, clinical trial patient populations often differ from the general patient populations owing to strict inclusion criteria, which may limit the applicability of our study conclusions. However, the clinical trials analyzed in this model had permissive inclusion criteria, including adult age, confirmed pathologic diagnosis, and only excluded patients with severe comorbidities, which is a reasonable representation of the true patient population. Furthermore, comparisons of clinical trials and population-based observational data show that clinical trial outcomes are generally reliable despite restrictive inclusion criteria.^43^ Seventh, we used Surveillance, Epidemiology, and End Results data, which lack variation status information, to estimate long-term survival beyond the follow-up of the clinical trials.

## Conclusions

In this economic evaluation, we present a Markov model comparing TGT with tailored dose selection of imatinib to empirical imatinib therapy. The model suggests that TGT-directed therapy is a cost-effective intervention compared to empirical imatinib. These findings support widespread adoption of standard genetic testing for newly diagnosed patients with metastatic GIST.

## Notes

Supplement. **eFigure 1.** Validation of Progression-Free Survival Transition Probabilities **eFigure 2.** Validation of Overall Survival Transition Probabilities **eTable 1.** Cost-Effectiveness Results for Different Assumptions About SDs for Parameters With Unknown Distributions **eTable 2.** One-Way Sensitivity Analysis
