# Supplementary material for: Cost-effectiveness Analysis of Genetic Testing and Tailored First-Line Therapy for Patients With Metastatic Gastrointestinal Stromal Tumors
Source: JAMA Netw Open. 2020 Sep 28;3(9):e2013565. doi: 10.1001/jamanetworkopen.2020.13565 (PMC7522695; doi:10.1001/jamanetworkopen.2020.13565)
Supplement: Supplement. — eFigure 1. Validation of Progression-Free Survival Transition Probabilities eFigure 2. Validation of Overall Survival Transition Probabilities eTable 1. Cost-Effectiveness Results for Different Assumptions About SDs for Parameters With Unknown Distributions eTable 2. One-Way Sensitivity Analysis [file jamanetwopen-e2013565-s001.pdf]

Supplemental Online Content

Banerjee S, Kumar A, Lopez N, et al. Cost-effectiveness analysis of genetic testing and tailored first-line therapy for patients with metastatic gastrointestinal stromal tumors. *JAMA Netw Open*. 2020;3(9):e2013565. doi:10.1001/jamanetworkopen.2020.13565

eFigure 1. Validation of Progression-Free Survival Transition Probabilities
eFigure 2. Validation of Overall Survival Transition Probabilities
eTable 1. Cost-Effectiveness Results for Different Assumptions About SDs for Parameters With Unknown Distributions
eTable 2. One-Way Sensitivity Analysis

This supplemental material has been provided by the authors to give readers additional information about their work.

© 2020 Banerjee S et al. *JAMA Network Open.*

eFigure 1. Validation of Progression-Free Survival Transition Probabilities

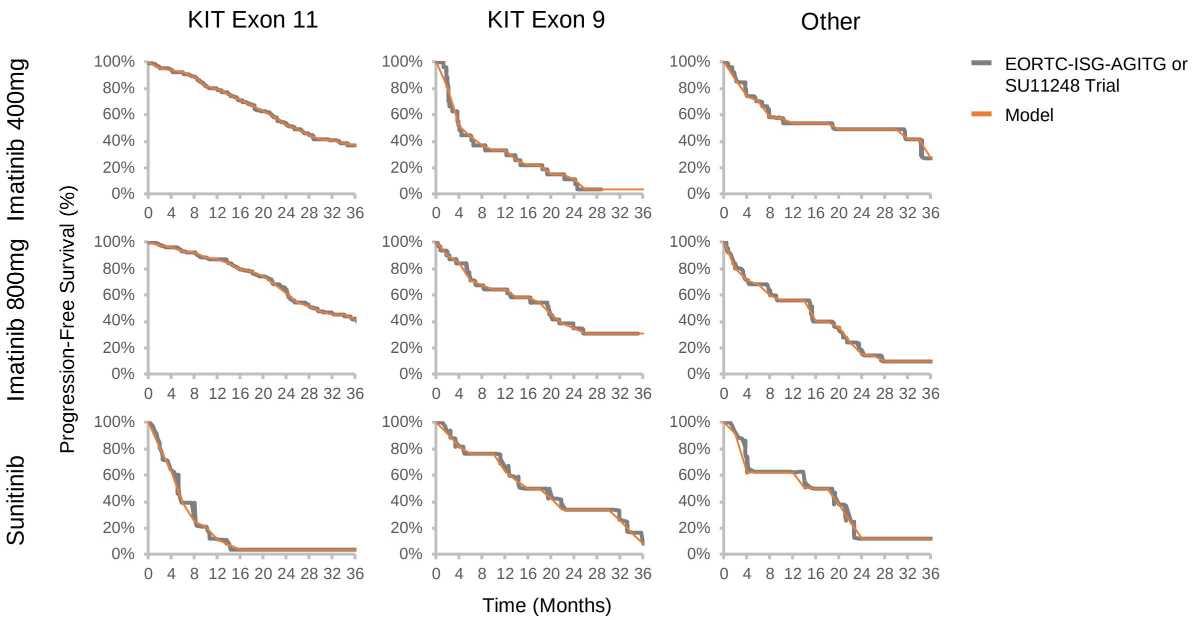

Extrapolated values from EORTC-ISG-AGITG or SU11248 trial data for each genomic subpopulation and each treatment (gray). Model transition probabilities (orange).

© 2020 Banerjee S et al. *JAMA Network Open.*

eFigure 2. Validation of Overall Survival Transition Probabilities

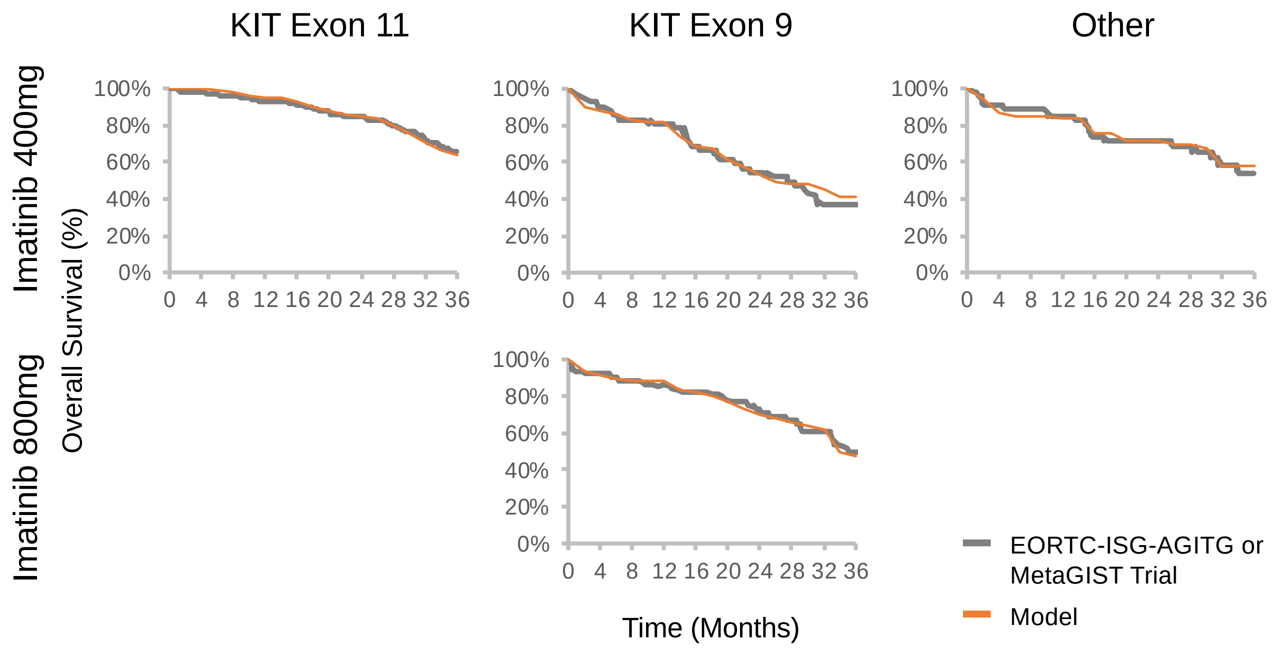

Extrapolated values from EORTC-ISG-AGITG or MetaGIST trial data for each genomic subpopulation and each first-line treatment (gray). Model transition probabilities (orange).

© 2020 Banerjee S et al. *JAMA Network Open.*

eTable 1. Cost-effectiveness results for different assumptions about standard deviations for parameters with unknown distributions

Scenario	Value	Percentage Cost effective at $100,000/QALY (100,000 iterations)
Standard deviation for parameters with unknown distributions	0.1	78.5
	0.15	72.7
	0.2*	69.9
	0.30	67.8
	0.40	61.7

Abbreviations: QALY = quality-adjusted life-year.
*Base case assumption

© 2020 Banerjee S et al. *JAMA Network Open.*

eTable 2. One-way sensitivity analysis

Parameter	Parameter Range*	ICER†
Cost of sequencing (one time)	1460 to 5838	78009 to 120415
Costs of imatinib 400 mg (per year)	28845 to 115380	73640 to 129166
Costs of Sunitinib (per year)	43363 to 173452	65431 to 158625
Costs of best supportive care (per year)	4702 to 18806	90313 to 95811
Other medical costs associated with imatinib (per year)	1158 to 4630	92575 to 91291
Other medical costs associated with sunitinib (per year)	1672 to 6688	90946 to 94544
Cost of adverse events related to imatinib (per month)	148 to 592	92128 to 92183
Costs of adverse events related to sunitinib (per month)	80 to 320	92136 to 92168
Cost of disease progression (per year)	23274 to 93096	110243 to 55955
Health utility of metastatic GIST (per year)	0.75 to 1	180518 to 75227
Health utility of disease progression (per year)	-0.14 to -0.10	88285 to 96362
Probability of adverse event related to imatinib 400 mg (per month)	0.026 to 0.040	92165 to 92130
Probability of adverse event related to imatinib 800 mg (per month)	0.052 to 0.078	92122 to 92169
Probability of adverse event related to sunitinib (per month)	0.025 to 0.037	92143 to 92151

Abbreviations: QALY, quality-adjusted life-year; ICER, incremental cost-effectiveness ratio.
*We varied costs from half the base case value to double. We varied health utilities and probabilities by 20% from the base case values.

© 2020 Banerjee S et al. *JAMA Network Open.*
